# Glancing Angle Deposited Nanostructured Tellurium Layer Against Dendrite Formation and Side Reactions in Aqueous Zn-Ion Battery Anode

**DOI:** 10.3390/nano15120952

**Published:** 2025-06-19

**Authors:** Salim Hussain, S. M. Sayem, Assem Basurrah, Tahany Rashed, Fumiya Watanabe, Noureen Siraj, Tansel Karabacak

**Affiliations:** 1 School of Physical Sciences, University of Arkansas at Little Rock, Little Rock, AR 72204, USA; shussain@ualr.edu (S.H.); ssayem@ualr.edu (S.M.S.); aobasurrah@ualr.edu (A.B.); tnrashed@ualr.edu (T.R.); nxsiraj@ualr.edu (N.S.); 2 Applied College, University of Jeddah, Jeddah 23890, Saudi Arabia; 3 Center for Integrative Nanotechnology Sciences, University of Arkansas at Little Rock, 2801 South University Avenue, Little Rock, AR 72204, USA; fxwatanabe@ualr.edu

**Keywords:** nanostructured tellurium layer, GLAD, Zn anode, surface modification, Zn-ion battery

## Abstract

Aqueous zinc ion batteries (AZIBs) have considerable potential for energy storage owing to their cost-effectiveness, safety, and environmental sustainability. However, dendrite formation, hydrogen evolution reaction (HER), and corrosion of the bare zinc (B-Zn) anode tremendously impact the performance degradation and premature failure of AZIBs. This study introduces a glancing angle deposition (GLAD) approach during the sputtering process to fabricate tellurium nanostructured (TeNS) at the zinc (Zn) anode to avoid the aforementioned issues with the B-Zn anode. Three different deposition times (5, 10, and 30 min) were used to prepare TeNS at the Zn anode. The morphology, crystallinity, composition, and wettability of the TeNSs were analyzed. The TeNSs served as hydrophilic sites and a protective layer, facilitating uniform Zn nucleation and plating while inhibiting dendrite formation and side reactions. Consequently, the symmetric cell with TeNS deposited on the Zn anode for 10 min (Te@Zn_10 min) demonstrated an enhanced cycling stability of 350 h, the lowest nucleation overpotential of 10.65 mV at a current density of 1 mA/cm^2^, and an areal capacity of 0.5 mAh/cm^2^. The observed enhancement in the cycling stability and reduction in the nucleation overpotential can be attributed to the optimal open area fraction of the TeNSs on the Zn surface, which promotes uniform Zn deposition while effectively suppressing side reactions.

## 1. Introduction

The escalating global consumption of fossil fuels is a significant contributor to the intensification of global warming [1]. Projections indicate that if this trend continues unabated, the Earth’s temperature could reach unprecedented levels not seen in two million years by the end of the twenty-first century [2,3]. Decreasing reliance on fossil fuels and facilitating the sustainable development of renewable energy resources is vital for ensuring global sustainability. Solar, geothermal, wind, bioenergy, and hydrothermal energy are some of the resources that have gained significant interest because of their potential in mitigating the effects of climate change [4,5,6]. However, the intermittent nature of these resources is a challenge in achieving a stable supply of energy. Hence, the development of effective and reliable energy-storage systems is of prime concern [7].

Recently, rechargeable aqueous batteries have been extensively studied owing to their eco-friendliness, inherent safety, low cost, quick charging capabilities, and high power density; thus, they are potential candidates for large-scale stationary energy-storage applications [8]. Among them, aqueous zinc-ion batteries (AZIBs) are of significant interest because of the merits of the Zn anode with desirable properties, including a high volumetric capacity (5855 mAh/cm^3^), high gravimetric capacity (820 mAh/g), and low reduction potential (−0.762 V versus the reference hydrogen electrode) [9,10]. Moreover, Zn is abundant, inexpensive, and has low toxicity, making AZIBs a viable alternative to traditional lithium-ion batteries (LIBs). Although LIBs have extensively transformed portable electronics and electric vehicles owing to their high-power density and excellent cycling stability [11,12], issues of cost, complex fabrication, safety, environmental concerns, and the limited availability of lithium resources have enhanced the quest for more sustainable energy storage systems [13,14]. AZIBs address many of these challenges; however, their extensive application has been hindered by several major challenges. The deposition of Zn ions is impeded by slow kinetics, primarily attributed to the solvation sheath formed by six water molecules, which hinders electron tunneling and the reduction process [15]. Consequently, dendrite formation and severe side reactions such as hydrogen evolution reaction (HER) and corrosion occur, leading to reduced cycle life and ultimately hindering the broader adoption and commercialization of AZIBs [16].

Various strategies have been investigated to enhance the performance of AZIBs, such as Zn surface modification, structure design, electrolyte engineering, and separator optimization [17,18,19,20,21,22]. Among them, the surface modification of Zn electrodes has been demonstrated to be an effective and simple way to enhance the performance of Zn anodes [23,24]. Protective layers have been extensively employed in LIBs and sodium-ion batteries (SIBs) to inhibit metallic dendrite growth because of their mechanical strength and resemblance to solid electrolyte interphase (SEI) layers. Inspired by such developments, analogous design strategies have been pursued for Zn anodes, including physical protection and electrochemical environment regulation [25,26,27]. Recent studies have investigated a variety of materials, from insulators to semimetals and metals, to stabilize and improve the electrochemical efficiency of Zn anodes. Some promising developments involve a nanoporous CaCO_3_ buffer layer [28], carbon films [29], polyimide interfacial layers [30], 0D metal/2D metal carbides interfacial layers [31], Au nanoparticle seeding [32], CrN coatings [33], and ZnSe layers [20], which are shown to effectively inhibit dendrite growth, avoid side reactions, and enhance cycle life, with some demonstrating excellent durability exceeding 3700 h. Other protective layers, such as ZrO_2_, TiO_2_, sodium Zn phosphate (NZP), Ag, and Bi_2_Te_3_, have also shown potential for improving Zn anode cycle life [34,35,36,37,38]. However, most of these materials serve as insulating interphases, are synthesized by wet chemistry methods, and are subsequently coated onto the Zn surface, which impedes zinc ion transport through the pores and fractures. Therefore, investigating a zincophilic and binder-free layer with high Zn^2+^ ion conductivity is crucial for controlling the Zn development.

Owing to their stable physical properties, various chalcogens and chalcogenides have been explored as protective layers for Zn anodes. Tellurium (Te), a metalloid, is a promising candidate as a protective film for Zn anodes in AZIBs, owing to its zincophilic nature, high electrical conductivity (2 × 10^2^ S/m), and notable surface energy of 355 mJ/m^2^ [39,40,41]. This high surface energy enhances the wettability of the Zn anode, and its high electrical conductivity improves electrochemical activity, increases the concentration of active species, and accelerates the kinetics during Zn plating and stripping processes. Moreover, the chemical stability of Te inhibits the rate of side reactions at the Zn anode [42]. Furthermore, Te nanostructures (TeNSs) promote fast electron transfer between the charging and discharging of ZIBs. Fu et al. designed a protective layer for a Te-hybridized core–shell metal–organic framework using a multistep solution-based process [43]. Lee et al. used a fast and facile surface treatment method using a Te complex to form Te nanoparticles on Zn surfaces [44]. However, these investigations primarily employed chemical methodologies without comprehensive optimization of growth parameters. Furthermore, the complex interplay between protective layer design, coating thickness, and long-term Zn anode performance remains unclear. Thus, there is still a need for a more straightforward yet effective Te deposition approach that eliminates the use of hazardous chemicals while facilitating precise control over film growth processes.

**Figure 1:**
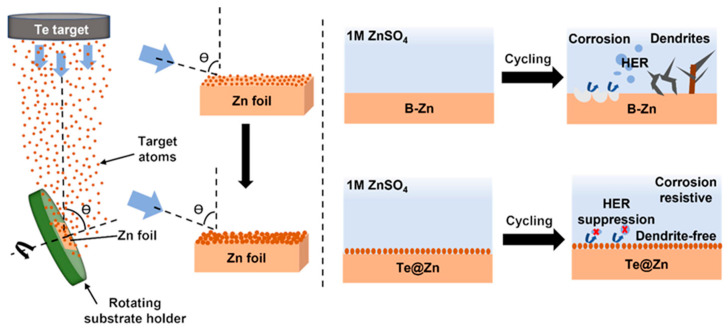
Schematic presentation of anode preparation process by depositing Te on Zn foil using GLAD technique (**left**), and mechanism of Zn stripping/plating on B-Zn and Te@Zn anode (**right**).

Glancing angle deposition (GLAD) is a scalable and versatile methodology for the fabrication of three-dimensional nanostructures that allows for precise control of nanostructure thickness and uniformity. GLAD offers an eco-friendly pathway for the synthesis of nanostructures, unlike other techniques such as chemical vapor deposition (CVD) and wet-chemical synthesis, which generally involve the utilization of toxic chemicals, solvents, and additives [45,46,47]. GLAD typically adheres to the Volmer–Weber growth mode, where adatoms exhibit greater reactivity with each other than with the substrate, resulting in the development of three-dimensional islands owing to increased adatom-adatom interaction [48,49]. Initially, nucleation occurs randomly, but the subsequent growth has a preferred orientation and is a competitive process because of the GLAD design. During GLAD deposition, the substrate holder can be adapted to facilitate both rotational and tilting movements (Figure 1). This process exploits the shadowing effect, wherein material particles approaching the surface at an oblique angle are selectively deposited on the uppermost regions of the surface features at greater elevations. This selective growth results in the development of discrete nanostructures [50,51,52,53]. In this study, we present for the first time a GLAD approach in a DC magnetron sputtering system to modify the surface of Zn foil by coating it with TeNSs. Detailed physical analyses, including dimensions, morphology, composition, crystallinity, and wettability of GLAD TeNSs at different deposition times, were performed. The electrochemical performances of the newly developed electrodes were evaluated by fabricating AZIB coin cells. This study presents an effective, scalable, and environmentally sustainable method to prepare a nanostructure layer at the surface of the Zn anode to attain an improved life cycle with enhanced electrochemical performance of the AZIB.

## 2. Materials and Methods

### 2.1. Materials and Chemicals

The Te target with a 2-inch diameter, 0.125-inch thickness, and 99.999% purity was obtained from Plasmaterials Inc. (Livermore, CA, USA). Zn foils of 0.1 mm thickness and 99.9% purity were purchased from MSE Supplies LLC., Tucson, AZ, USA. The P3000 sandpaper was purchased from Amazon.com (Seattle, CA, USA). Acetone and isopropanol were obtained from Fisher Scientific (Waltham, MA, USA). Ultrapure DI water of 18.2 MΩ.cm resistivity was produced in the laboratory using a combination of the Micropure ST, Mili-Q Academic systems eBay (San Jose, CA, USA), Whatman glass microfiber grade GF/D prefilter discs separator of 2.7 μm pore size, and zinc sulfate heptahydrate (ZnSO4.7H2O) powder to prepare electrolyte, which were obtained from Sigma-Aldrich (St. Louis, MO, USA).

### 2.2. Anode Preparation

Zn foils were sectioned into four 5 × 5 cm^2^ pieces and meticulously polished manually using sandpaper to remove organic contaminants and native oxides. Subsequently, the foils were ultrasonicated in acetone, isopropanol, and deionized water for 10 min each. The cleaned foils were desiccated using a nitrogen stream. One of the desiccated foils was designated as the “B-Zn” anode, which was directly transferred for physical characterization and electrochemical tests. The remaining foils were used for the fabrication of three different Te-coated anodes via GLAD, each sample having a different Te deposition time (5, 10, and 30 min). For GLAD, we utilized a custom-made sputter deposition unit. Argon (Ar) of purity 99.999% was employed as the carrier gas, and a Te sputter target served as the deposition material source. The base pressure was ~2 × 10^−6^ mbar, and argon pressure was ~4.0 × 10^−3^ mbar with a 10 sccm Ar flow rate. The DC power used was 25 W and the distance between the substrate holder and the Te source was approximately 20 cm. The nanostructures were grown at a fixed deposition angle (ɵ) value of 84° relative to the substrate normal, with an azimuthal rotation of the substrate at a speed of about 22 rpm around the central normal axis of the substrate surface. The deposition angle of 84° was selected based on previous studies that suggest that increasing the deposition angle significantly reduces the feature size of the resulting material [54,55]. At such oblique angles, a geometrical “shadowing effect” occurs, whereby the incoming vapor preferentially deposits onto elevated surface regions that are more directly exposed. This anisotropic deposition results in a preferential growth mechanism that facilitates the formation of well-separated and uniform nanostructures. Additionally, continuous rotation of the substrate during the GLAD process plays a critical role in achieving uniform and vertically aligned nanostructures. To determine the nominal thickness of the TeNS layer on Zn substrates, we carried out independent depositions on quartz crystal microbalance (QCM) sensors affixed to the substrate holder. The mass of the deposited material was calculated from the shift in the crystal’s resonant frequency, applying the Sauerbrey equation [56,57]. Using the deposition rate derived from this calibration and considering the estimated amount of open area (i.e., gaps; see Section 2.5 for details), the corresponding TeNS thicknesses for deposition durations of 5, 10, and 30 min were estimated to be approximately 27 nm, 52 nm, and 153 nm, respectively. Based on these nominal thickness values, we calculated the average deposition rate as ~0.87 Å/s. These samples were subsequently labeled Te@Zn_5 min, Te@Zn_10 min, and Te@Zn_30 min, corresponding to their respective deposition durations. The substrate was rotated continuously at a fixed rate throughout the process. In addition, a Te thin film was also deposited on the Zn foil with the deposition time of 60 min but at the normal incidence flux for Raman analysis and comparison with the TeNSs-fabricated anodes. To ensure minimal topographical variation between the control and Te-coated surfaces, Zn plates were sequentially polished using progressively finer grit sandpapers to achieve a smooth and uniform surface finish. Following surface preparation, a very thin film of tellurium (~47 nm) was deposited onto the polished Zn substrates using DC magnetron sputtering under controlled conditions. This setup enabled a direct comparison of wettability influenced predominantly by surface chemistry rather than roughness.

### 2.3. Material Characterizations

SEM (JOEL USA Inc. JSM-7000F, Peabody, MA, USA) operated at 15 kV was used to analyze the morphology of the Zn foil samples. The SEM images shown in Figure 1 were captured at a magnification of 75,000×. The magnification for Supplementary Figure S1 was 50,000×. The crystal structure and composition of the ZnO nanorods were analyzed by X-ray diffraction (XRD, Rigaku Miniflex Rigaku America Co., The Woodlands, TX, USA) with Cu Kα radiation (λ = 1.5418 Å) and Raman scattering spectra (LabRam HR800, HORIBA Instruments Inc., Irvine, CA, USA) at a laser wavelength of 514.5 nm. X-ray photoelectron spectroscopy (XPS; K-Alpha, Thermo Fisher Scientific, Waltham, MA, USA) was used to investigate the surface chemical states of the samples. The wettability of the chemically modified Zn foils was investigated using a VCA Optima goniometer (AST Products, Inc., Billerica, MA, USA).

### 2.4. Electrochemical Characterization

The 2032 symmetric battery coin cells were fabricated using two B-Zn or Te@Zn electrodes in air, where a cellulose-based fiber was used as a separator, and a 1M ZnSO_4_ was utilized as an electrolyte. The electrochemical cycling performance (galvanostatic charge discharge (GCD) profile) of symmetric cells was recorded by Arbin battery test equipment (Arbin Instruments, College Station, TX, USA). The electrochemical workstation (“Interface 1010 Potentiostat/Galvanostat/ZRA” Gamry Instruments, Warminster, PA, USA) was used to record electrochemical impedance spectroscopy (EIS) and linear sweep voltammetry (LSV). EIS was performed at a 10 mV potential amplitude and a frequency range of 0.1–100,000 Hz on the fabricated symmetric cells. The corrosion and HER behaviors of the samples were investigated using a three-electrode setup in the presence of 1M ZnSO_4_ electrolyte using LSV measurement at 5 mV/s, B-Zn or Te@Zn as the working electrode, a Pt wire counter electrode, and an Ag/AgCl reference electrode.

### 2.5. Image Processing

Open-source Core Fiji plugins bundled with the Fiji distribution of ImageJ 1.54p were utilized to obtain the open-area fraction of the Te-deposited samples from the top-down SEM images. The open-area fraction was defined as the areal fraction of the gaps between the TeNSs. SEM micrographs were imported into ImageJ and converted to an 8-bit format by selecting “Type” under “Image” and then “8-bit”. The micrograph scale bar was traced using the line tool to calibrate the image under the “Analyze” → “Set Scale” function. The micrograph contrast was initially optimized with the “Enhance Local Contrast” filter. The size distribution of the nanoparticles was determined manually by measuring the diameter of each particle in multiple directions and subsequently calculating the mean and standard deviation. For the porosity measurement, the full image was divided into numerous smaller regions with similar contrast, each of which was duplicated. These duplicated images then underwent the same procedure, the “Image” → “Adjust” → “Adjust Threshold” function in the dark background mode, to develop a black/white contrast between the individually resolved nanopores and the TeNS. The number and area of open regions between the Te islands were obtained using the “Analyze” → “Analyze Particles” function with a manual minimum area of 1 nm^2^ and a roundness factor of 0.2 to mitigate the effects of noise. It is important to acknowledge that the results obtained through the aforementioned method are confined to the “visible” open areas as determined by the SEM’s spatial resolution, potentially excluding any open areas that fall below this resolution threshold.

## 3. Results and Discussion

A schematic representation of our strategy for preventing dendrite formation and side reaction suppression on the Zn electrode is shown in Figure 1 (right). On the Zn foil, TeNSs anodes were fabricated with varying deposition times using GLAD through a single-step DC magnetron sputtering method. TeNSs provide an electrode-electrolyte contact that is believed to successfully shield the Zn metal from dendrite growth, corrosion, and HER and facilitate rapid Zn^2+^ diffusion as well.

**Figure 2:**
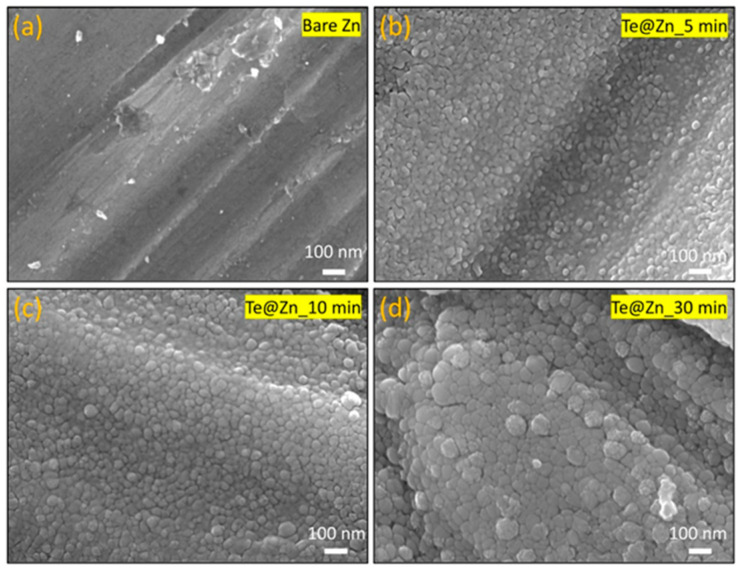
Scanning electron microscopy (SEM) images of Bare Zn (**a**) before GLAD, and (**b**–**d**) after 5, 10, and 30 min of Te deposition, respectively, using GLAD.

**Figure 3:**
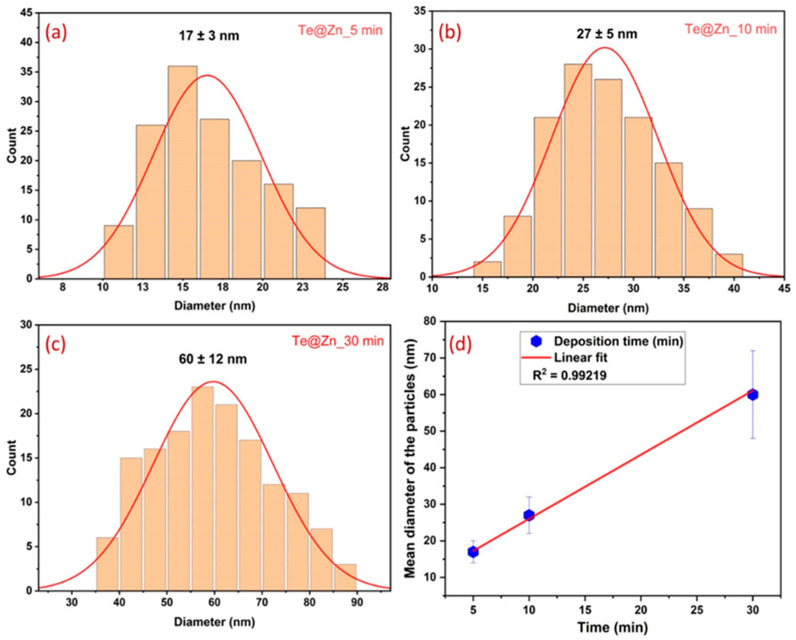
Normal size distribution of the Te nanoparticles after (**a**) 5 min, (**b**) 10 min, and (**c**) 30 min of Te deposition using GLAD; (**d**) linear fit of mean diameter of the deposited particles vs. deposition time.

The SEM images show the surfaces of the Zn foils prior to (Figure 2a) and following (Figure 2b–d) Te deposition using GLAD. The B-Zn foil exhibited only microroughness owing to sandpaper polishing without any observable nanofeatures. After Te deposition on Zn at different periods (Te@Zn_5 min, Te@Zn_10 min, and Te@Zn_30 min), quasi-spherical Te particles of variable sizes were observed on the Zn surfaces. This transformation demonstrates the effectiveness of the GLAD technique in synthesizing nanostructured surfaces with controlled particle size deposition. Particle size distribution analysis, as shown in Figure 3, depicts a gradual increase in the mean particle size with respect to the deposition time. Specifically, the particles exhibited average diameters of 17 ± 3 nm, 27 ± 5 nm, and 60 ± 12 nm for Te@Zn_5 min, Te@Zn_10 min, and Te@Zn_30 min, respectively, as illustrated in Figure 3a–c. Figure 3d shows a linear increasing trend in the mean particle diameter with deposition time, suggesting a consistent and predictable growth rate of Te nanoparticles on the Zn substrate. However, the very short deposition times at low deposition rates, Te was initially deposited as discrete islands on the Zn foils instead of as a complete continuous film. The calculated open areas between the Te islands were 7.2, 3.2, and 1.7%, respectively, which indicates that with longer deposition times, the Te coverage on the Zn surface was more continuous and uniform. The uniform size distribution and optimal isolation of Te particles function as zincophilic sites and protective layers for the Zn surface, thereby facilitating homogeneous Zn deposition and ensuring a consistent Zn^2+^ flux [58,59].

**Figure 4:**
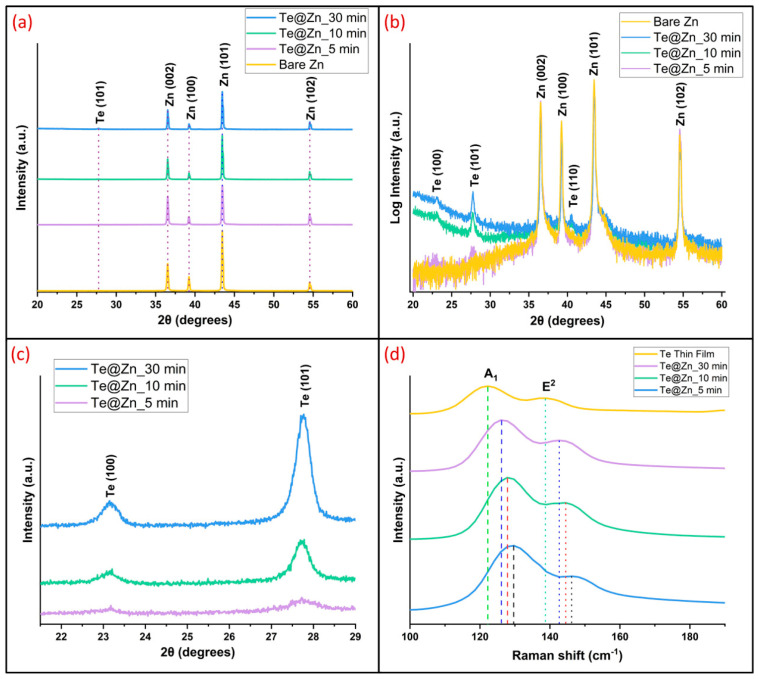
(**a**) X-ray diffraction (XRD) patterns on a linear scale for Bare Zn and Te@Zn samples with 5, 10, and 30 min deposition, (**b**) corresponding XRD patterns on a logarithmic scale, (**c**) magnified view highlighting Te (100) and Te (101) reflections for Te@Zn (5, 10, and 30 min), and (**d**) Raman spectra for Bare Zn and Te@Zn (5, 10, and 30 min).

XRD analysis was performed at 2θ = 20–60° to examine the crystalline structure and composition of the B-Zn and Te-deposited Zn foils (Figure 4a). The B-Zn exhibited diffraction patterns at 2θ = 36.5°, 39.2°, 43.4°, and 54.6°, corresponding to the (002), (100), (101), and (102) planes of Zn, respectively [Crystallography Open Database entry 9012435]. An additional tiny peak appeared at 2θ = 27.7° for the Te@Zn_30 min and Te@Zn_10 min samples, which is attributed to the (101) plane of Te [Crystallography Open Database entry 9008522]. However, no such peak was observed for the Te@Zn_5 min sample in Figure 4a. The dominance of the substrate peaks in the pattern may be attributed to the near-perfect nature of the Zn single crystals. In contrast, Te peaks may appear significantly weaker because of the substantially thinner deposition layers [60]. For this reason, we plotted the XRD data on a logarithmic scale in Figure 4b, where, in addition to the clear peak of (101), two additional peaks at 2θ = 23.13° and 40.54° corresponding to the (100) and (110) planes of Te are observed in all Te-deposited samples [Crystallography Open Database entry 9008522]. Additionally, to determine the relative intensities of the (100) and (101) planes, we performed XRD analysis at 2θ = 20–30° for a longer duration and smaller step size (Figure 4c). The intensity of the peaks exhibited a gradual upward trend, correlating with the longer deposition periods. The XRD patterns confirmed the good crystallinity of the trigonal Te crystal structure. Surface–electrolyte interaction is improved by crystalline Te because of its reduced lateral dimensions as compared to bulk Te. These characteristics are further enhanced by its enormous surface area, even distribution of nanoparticles, and intrinsic high conductivity. As a result, an improvement in the Zn^2+^ kinetics and ion flux on the Zn electrode surface is anticipated [61].

Raman spectroscopy was conducted on a Te thin film and the Te nanoparticles deposited on Zn foils using GLAD (Figure 4d). Two active Raman vibrational modes, A^1^ and E^2^, were observed at 122.3 and 138.7 cm^−1^ in the spectra of the bulk sample, which is ascribed to the crystalline phase of trigonal Te [62,63,64]. Compared to the Te nanoparticle samples, significant Raman shifts were observed in both modes as the dimensions of the Te particles decreased. Specifically, A^1^ and E^2^ modes exhibited a blueshift of 4 cm^−1^ for the 30 min sample relative to the bulk sample, whereas the modes demonstrated a shift of 1.8 nm^−1^ each for the 10 and 5 min samples compared to the 30 min sample. The peak shifts in the nanocrystals are attributed to the overlapping effects of quantum confinement and thermal effects [65]. According to the size distribution in Figure 2a–c, the Te nanoparticles synthesized in this study are fairly within the exciton-Bohr radius limit (50.97 nm) reported by Luke et al. [66]. However, even low laser power can increase the local temperature, which can change the bond lengths of atoms and consequently introduce Raman shifts [65]. The Raman spectra of all Te-deposited samples support the XRD data well, further confirming the crystallinity and composition of the TeNs on the Zn foils.

**Figure 5:**
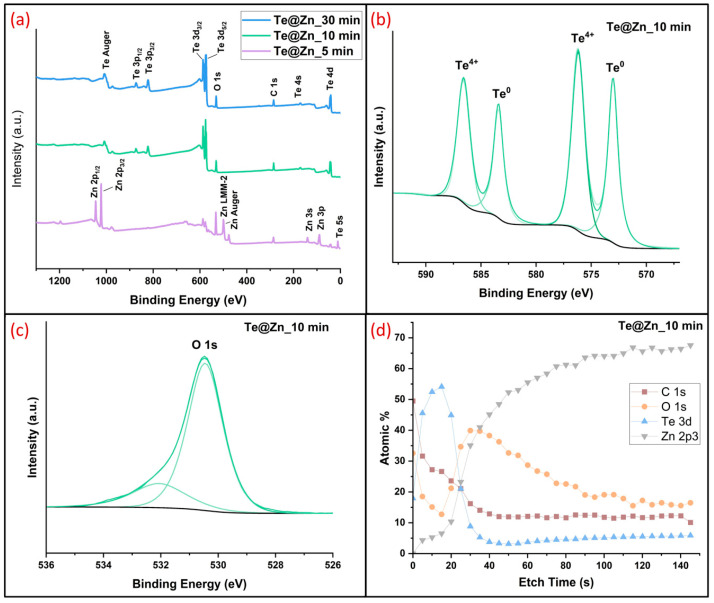
XPS analysis of Te nanostructures grown on Zn surface (**a**) the survey spectrum, (**b**) Te 3d scan, (**c**) O 1 s scan, and (**d**) depth profile of Te@Zn_10 min sample.

XPS was employed to analyze the surface chemical compositions of the Te-deposited samples. In addition to Te and Zn, the XPS survey spectrum (Figure 5a) reveals signals for oxygen (O) and carbon (C). The presence of C is commonly observed during XPS analysis, introduced from the laboratory environment, whereas O is likely due to the formation of native oxide on the Te surface. High-resolution XPS (Figure 5b) of the Te 3d spectra indicates that this region consists of four peaks. Zn peaks were detected in Te@Zn_5 min sample but not in the other samples, which is attributed to its shorter Te deposition time. This suggests that the TeNS layer in this sample was exceedingly thin. Conversely, Te@Zn_10 min and 30 min samples demonstrated higher intensities of Te peaks, specifically Te 3d3/4 and Te 3d5/2. The high-resolution XPS of the Te 3d spectra in this region shows four peaks, where 582.9 eV and 572.5 eV correspond to metalloid Te^0^, and 575.9 eV and 586.2 eV corelate to Te^4+^ state, which is attributed to native oxide formation (Figure 5b, Figures S2 and S3). Literature suggests that the oxidation of Te to form TeO_2_ results in a chemical shift of approximately 3.2 eV, which is evident in this case [67]. Owing to its high surface energy compared to the bulk, the nanostructured Te surface spontaneously oxidizes in air (approximately 1.5 nm in 24 h) [68]. Figure 5c shows high-resolution XPS of O 1 s spectra, which were resolved into two peaks. The peak with a lower binding energy at 530.5 eV is due to ions with the formal valence state of O_2_. While the higher binding energy component at around 532.3 eV is attributed to the presence of loosely bound oxygen on the surface of the film, such as CO_3_, H_2_O, or O_2_ [67]. Figure 5d, Figures S2 and S3 show the depth profile of the Te-deposited Zn samples. The majority of the surface oxygen was removed by sputter etching for 15 s, which indicates that the oxidation process for the Te nanoparticles occurred largely on the surface rather than in the core. The hump in the oxygen concentration, which was observed after the initial decrease, may have originated from the native tellurium oxide that remained unetched because of the micro-roughness of the underlying Zn foil surface [69], and/or from the native zinc oxide that could have been exposed after the removal of Te during etching. The formation of a native tellurium oxide layer may contribute to improved interfacial stability by acting as a passivation layer that suppresses undesirable side reactions. Additionally, it could facilitate Zn^2+^ ion transport, thereby supporting enhanced overall electrochemical performance. On the other hand, a tellurium oxide layer may be continuously formed and removed during battery charging and discharging, potentially rendering the native tellurium oxide layer irrelevant. This hypothesis requires detailed cyclic voltammetry studies to confirm.

**Figure 6:**
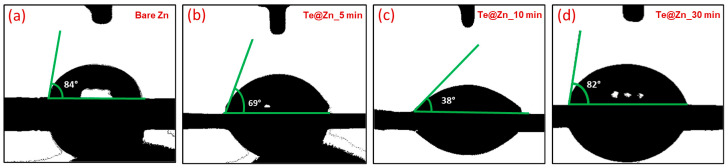
Contact angle analysis of (**a**) Bare Zn, (**b**) Te@Zn _5 min, (**c**) Te@Zn_10 min, and (**d**) Te@Zn_30 min using droplets of 1 M ZnSO_4_ aqueous electrolyte solution.

Contact angle (CA) measurements were performed in air using droplets of 1 M ZnSO_4_ aqueous electrolyte solution to assess the wettability characteristics of the four electrodes (Figure 6a–d). The CA of the B-Zn electrode (Figure 6a) was determined to be 84°, indicating slight hydrophilicity towards the electrolyte solution, which is consistent with the previous studies [30,32]. In addition to surface morphology, topography, surface chemistry, and composition are critical determinants of surface wettability and solid/liquid adhesion [70]. Figure 6b–d demonstrate a reduction in the CA values following Te deposition, which is consistent with prior findings [40]. The comparatively more hydrophilic nature observed in the Te-deposited samples relative to bare Zn (B-Zn) suggests that elemental Te and/or its native tellurium oxide layer may possess higher surface energy than Zn and its native zinc oxide counterpart. Although comprehensive and conclusive surface energy values for Te, Zn, and their respective oxides are limited or inconsistent in the existing literature, the wettability behavior observed in our experiments supports this interpretation. To isolate the effect of surface chemistry from topographical influences, we deposited an ultrathin Te layer on a relatively smooth Zn substrate, ensuring minimal differences in surface morphology between the Te-coated and uncoated Zn plates (Figure S4). CA measurements revealed a slight reduction in CA for the Te-coated surface, indicating enhanced hydrophilicity. According to Young’s equation and the Wenzel model, both surface energy and surface roughness govern wettability [71]. Given that surface roughness was held constant in this comparative study, the observed improvement in wettability is primarily attributed to the inherently higher surface energy of the Te coating. Regardless, Te@Zn_5 min sample exhibited a lower CA value (69°) than that of the B-Zn sample. In general, an increase in surface roughness tends to enhance the hydrophilicity of a surface that is already hydrophilic [72]. Thus, the CA of Te@Zn_10 min sample decreased further to 38° (Figure 6c). This enhanced hydrophilicity is likely due to the increased surface roughness of the Te layer compared to Te@Zn_5 min sample. Conversely, the CA of the Te@Zn_30 min sample (Figure 6d) increased to 82°, which is slightly less than that of the B-Zn sample, yet still relatively hydrophilic. This increase in the CA value can be attributed to the comparatively smoother surface of the Te@Zn_30 min electrode compared to other Te-coated Zn electrodes, as evidenced by the SEM images. Overall, Te@Zn_10 min electrode exhibited greater hydrophilicity than the other electrodes, resulting in a more uniform zinc ion flux by reducing the interfacial free energy between the electrode and aqueous electrolyte. This effectively inhibits dendrite growth by promoting dense Zn deposition and nucleation [73,74].

**Figure 7:**
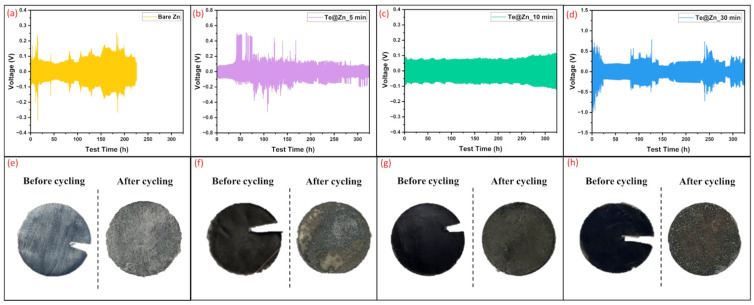
Galvanostatic charge/discharge (GCD) profiles for (**a**) Bare Zn, (**b**) Te@Zn_5 min, (**c**) Te@Zn_10 min, and (**d**) Te@Zn_30 min anode symmetric cells at 1 mA/cm^2^ and 0.5 mAh/cm^2^. Photographs of electrodes before and after cycling for (**e**) Bare Zn, (**f**) Te@Zn_5 min, (**g**) Te@Zn_10 min, and (**h**) Te@Zn_30 min samples.

GCD tests were conducted using symmetric cells (B-Zn||B-Zn or Te@Zn||Te@Zn) for each electrode to evaluate the effect of the TeNS layer deposited at varying durations on the Zn plating/stripping cycle performance. The cyclic performances of the B-Zn and Te-coated Zn symmetric cells were assessed at a current density of 1 mA/cm^2^ and a cycle time of 60 min, equating to an areal capacity of 0.5 mAh/cm^2^. The voltage–time profiles of the B-Zn, Te@Zn_5 min, and Te@Zn_30 min cells (Figure 7a,b,d) exhibited rapid voltage fluctuations from the initial cycles, indicating uncontrolled Zn dendrite formation. This dendrite growth penetrates into the separator and attaches to another electrode, causing the short circuits or battery failure. It is believed that the primary cause of dendrite growth was non-uniform Zn deposition during cycling, which led to the free diffusion of Zn^2+^ ions across the electrode surface. Consequently, Zn^2+^ ions easily migrate to energetically favorable sites for charge transfer, resulting in Zn^2+^ aggregation and acting as active sites for dendrite Zn nucleation [75]. In contrast, the Te@Zn_10 min cell demonstrated stable and minimal voltage polarization over approximately 350 h of cycling (Figure 7c). Although cycling was conducted beyond the test time range presented in Figure 7b–d, noticeable performance degradation occurred after approximately 350 h, particularly for electrodes other than Te@Zn_10 min. As such, voltage profiles beyond this period are not shown. This exceptional cycling stability is likely attributed to the hydrophilic nature of the Te electrode deposited for 10 min duration, which promotes uniform Zn ion flux [76]. After cycling, B-Zn, Te@Zn_5 min, and Te@Zn_30 min electrodes appeared worn and tattered (Figure 7e–h), which is most likely due to the development of large dendrites or protrusions combined with a dramatic redistribution of Zn mass. Nevertheless, the Te@Zn_10 min electrode maintained a highly integrated disc shape (inset photograph of Figure 7d) with a comparatively homogeneous surface morphology after cycling.

**Figure 8:**
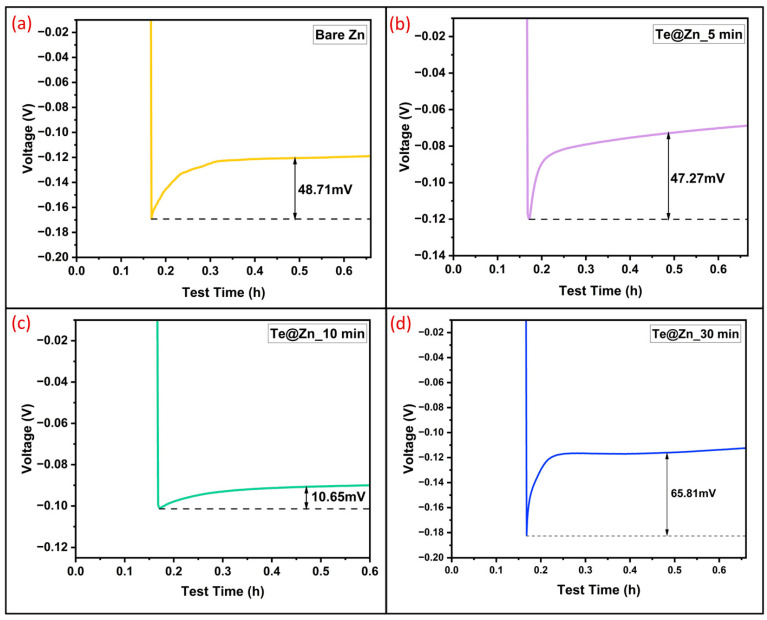
Nucleation overpotential of (**a**) Bare Zn, (**b**) Te@Zn_5 min, (**c**) Te@Zn_10 min, and (**d**) Te@Zn_30 min anode symmetric cells at 1 mA/cm^2^ and 0.5 mAh/cm^2^.

In addition, the nucleation overpotential was determined by selecting and amplifying the initial time–voltage curves of the cells. Figure 8a–d shows the time–voltage plots for the first discharge cycle. The nucleation overpotentials of B-Zn, Te@Zn_5 min, and Te@Zn_30 min cells were 48.71, 47.27, and 65.81 mV, respectively. Conversely, the nucleation overpotential of the Te@Zn_10 min cell was only 10.65 mV at 1 mA/cm^2^ and 0.5 mAh/cm^2^, demonstrating an optimal TeNS layer that reduces the energy barrier for Zn nucleation/dissolution at the electrode/electrolyte interface. The lower nucleation overpotential of the Te@Zn_10 min cell is also attributed to the more hydrophilic nature of this electrode [77]. The Zn plating/stripping reactions were clearly influenced by the overpotentials, which are related to the kinetics of Zn stripping/plating reactions as well as the Zn^2+^ and electron transfer resistances [78]. Zn^2+^ and electron transfer resistance are typically considered to be inherent characteristics of the electrodes and electrolytes used. The size as well as size distribution of plating nuclei, surface tension, nucleation, and other factors affect the kinetics of Zn stripping/plating [79,80].

**Figure 9:**
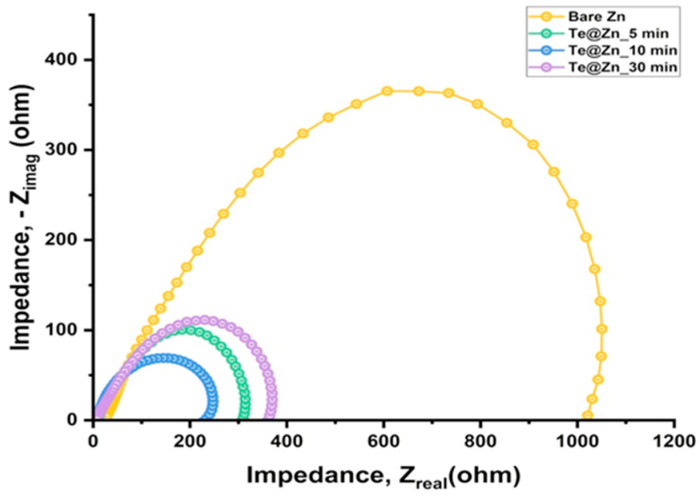
EIS profiles for symmetric cells assembled with Bare Zn and Te@Zn electrodes (5, 10, and 30 min deposition).

EIS was performed on symmetric cells to examine the charge transfer kinetics of the Zn^2+^ at the electrode/electrolyte interface. In Figure 9, the smallest semicircle is associated with the Te@Zn_10 min symmetric cell, which exhibited the lowest charge-transfer resistance value of 235 Ω. This value is approximately one-quarter of the charge-transfer resistance observed in the B-Zn symmetric cell, which is 1021 Ω. Moreover, the Te@Zn symmetric cell also manifested a greater charge transfer efficiency due to TeNS than the B-Zn symmetric cell [81]. In contrast, resistance values for Te@Zn_5 min and Te@Zn_30 min cells were 312 Ω and 367 Ω, respectively. These findings suggest that the 10 min deposited TeNSs enhance deposition kinetics, enabling homogeneous Zn plating. They may also contribute to reduced nucleation overpotential observed in the Te@Zn_10 min symmetric cell [30,82].

**Figure 10:**
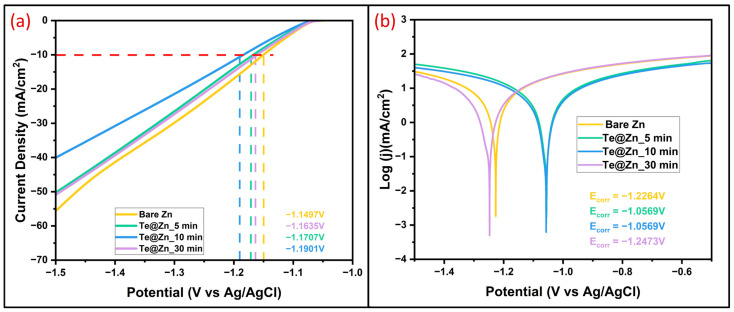
(**a**) LSV curves of B-Zn and Te@Zn samples (deposited for 5, 10, and 30 min) at a scan rate of 5 mV/s in 1 M ZnSO_4_ electrolyte, and (**b**) Tafel polarization curves of Bare Zn and Te@Zn electrodes.

The thermodynamic instability of Zn in aqueous solutions leads to side reactions, including corrosion and HER [83,84]. These undesirable side effects possibly contribute to performance degradation and battery failure by exacerbating dendrite Zn growth and by-product accumulation on the Zn surface. A three-electrode setup was employed to assess the HER and anticorrosion behavior of the prepared anodes in a 1M ZnSO_4_ electrolyte. Compared to B-Zn, Te@Zn_5 min, and Te@Zn_30 min, the cathodic current density (j) response increases slowly for Te@Zn_10 min (Figure 10a). The onset HER potentials (Figure 10a) for B-Zn, Te@Zn_5 min, Te@Zn_10 min, and Te@Zn_30 min are −1.1498, −1.1707, −1.1636, and −1.1901 V, respectively. The Te@Zn_10 min electrode exhibited the lowest potential magnitude, indicating minimal hydrogen evolution. This is attributed to its low nucleation overpotential and high hydrophilicity. Furthermore, the Tafel plot (Figure 10b) indicated that the corrosion potentials of B-Zn, Te@Zn_5 min, Te@Zn_10 min, and Te@Zn_30 min were −1.2264, −1.0569, −1.0569, and −1.2473, respectively, indicating that the Te@Zn_10 min electrode has superior anti-corrosion properties. These results indicate that in a 1M ZnSO_4_ electrolyte, the Te nanostructure protection layer with 10 min of deposition effectively inhibits the HER and enhances anti-corrosion capacity [40,70,72]. Furthermore, the overpotential and cyclic performance of the Te@Zn_10 min symmetric cell were significantly superior to those reported in the majority of published studies (Table 1). It demonstrates that TeNS-layer using the GLAD approach is a promising method to increase the cycling stability as well as to improve the overall performance of AZIBs in real applications. However, the Te@Zn_10 min electrode symmetric cell exhibited stable cycling for approximately 350 h at 1 mA cm^−2^, highlighting the effectiveness of the TeNS coating in enhancing interfacial stability. While these results are promising, further optimization of the tellurium nanostructure growth conditions is necessary to improve long-term performance. Also, it is acknowledged that the coulombic efficiency analysis for symmetric cell and full cell measurements, which are essential for fully assessing practical viability, will be addressed in future work.

## 4. Conclusions

In conclusion, this study suggests a new and effective approach to enhance the performance of Zn metal anodes through surface modification using a TeNSs coating. To the best of our knowledge, this is the first time that Te has been deposited on Zn foils using GLAD for Zn anode fabrication. GLAD Te was deposited onto Zn foils utilizing a DC magnetron sputtering unit for various durations, and the SEM images revealed a uniform and isolated distribution of the TeNSs. XRD and Raman analyses were performed to investigate the crystallinity and chemical composition of the deposited TeNSs, respectively. The XPS analysis also provided the chemical composition information and revealed a thin native oxide layer on the sample surfaces. Electrochemical measurements were performed on the Te-coated samples on Zn foil and Zn free of Te deposition. The sample with 10 min Te deposition was more hydrophilic than naked Zn, with a higher Zn-ion flux, which exhibited inefficient dendrite growth and suppression in side reactions. Te@Zn_10 min symmetric cell exhibited improved electrochemical stability for up to 350 h at 1 mA/cm^2^ with 0.5 mAh/cm^2^ areal capacity as compared to the B-Zn anode, as well as 5 and 30 min Te-deposited electrode cells. Moreover, the Te@Zn_10 min anode also demonstrated a significantly reduced nucleation overpotential of 10.65 mV in comparison to the B-Zn anode. Furthermore, the Te@Zn_10 min symmetric cell displayed enhanced charge transfer kinetics for Zn^2+^ relative to the B-Zn symmetric cell. In a three-electrode setup, the Te@Zn_10 min electrode was found to inhibit side reactions such as corrosion and the HER to a great extent. This is due to the fact that the Te layer was strongly adhered to the Zn surface, passivating potential water molecule adsorption sites and thereby inhibiting corrosion and HER. Surface modification utilizing GLAD-deposited Te is an effective, straightforward, and scalable strategy that offers novel insights for enhancing the performance of Zn anodes. Additionally, this simple approach is expected to improve the stability of Zn anodes and provide a viable option for practical applications in AZIBs.

## Figures and Tables

**Table 1:** Comparison of Te@Zn symmetric cells with recent research on different Zn surface modification approaches.

Surface Modification Layers	Current Density (mA/cm^2^)	Areal Capacity (mAh/cm^2^)	Cycle Life (h)	Nucleation Overpotential (mV)	References
TeNS layer	1	0.5	~350	10.65	This work
Cu layer	1	1	~290	12	[85]
Au layer	0.25	0.05	~2000	111	[32]
TiO_2_ layer	1	1	~150	72.5	[35]
Ag layer	1	1	~1000	48	[37]

## Data Availability

Data are contained within the article and Supplementary Materials.

## Supplementary Materials

The following supporting information can be downloaded at: https://www.mdpi.com/article/10.3390/nano15120952/s1, Figure S1: Scanning electron microscopy (SEM) images of Zn foils after (a) 5, (b) 10, and (c) 30 min of Te deposition using glancing angle deposition (GLAD); Figure S2: High-resolution XPS of (a) Te 3d scan, (b) O 1s scan, and (c) depth profile of the Te@Zn_5 min sample; Figure S3: High-resolution XPS of (a) Te 3d scan, (b) O 1s scan, and (c) depth profile of the Te@Zn_30 min sample; Figure S4: Surface morphology and wettability of Zn plates with and without a ~47 nm Te thin film layer. Polished Zn plates before Te deposition: (a) low-magnification SEM, (c) high-magnification SEM, and (e) contact angle (CA) measurement. Polished Zn plates after Te deposition: (b) low-magnification SEM, (d) high-magnification SEM, and (f) CA measurement.
