# Supplementary material for: Glancing Angle Deposited Nanostructured Tellurium Layer Against Dendrite Formation and Side Reactions in Aqueous Zn-Ion Battery Anode
Source: Nanomaterials (Basel). 2025 Jun 19;15(12):952. doi: 10.3390/nano15120952 (PMC12196523; doi:10.3390/nano15120952)
Supplement: Supplementary file 1 [file nanomaterials-15-00952-s001.zip › nanomaterials-3603027-supplementary.pdf]

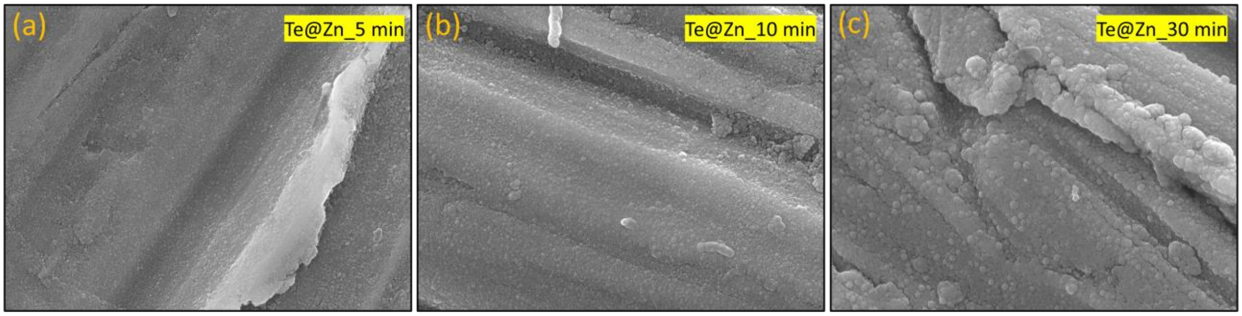


**Figure S1.** Scanning electron microscopy (SEM) images of Zn foils after (a) 5, (b) 10, and (c) 30 min of Te deposition using glancing angle deposition (GLAD).

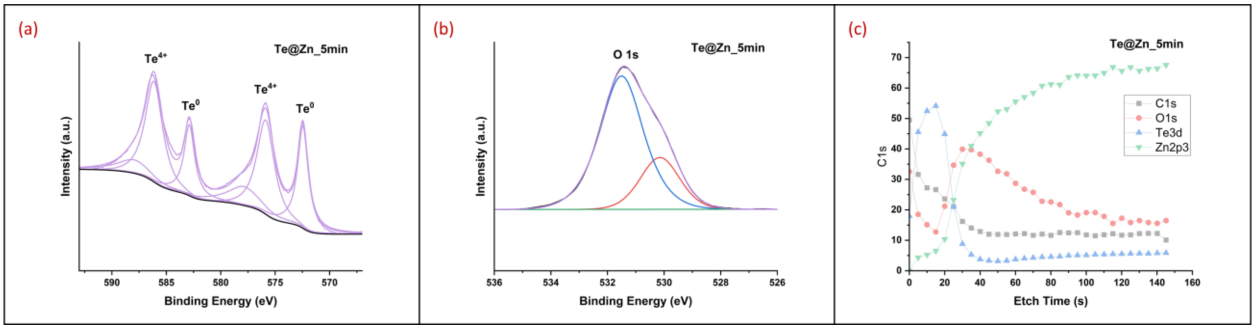


**Figure S2.** High-resolution XPS of (a) Te 3d scan, (b) O 1s scan, and (c) depth profile of the Te@Zn_5 min sample.

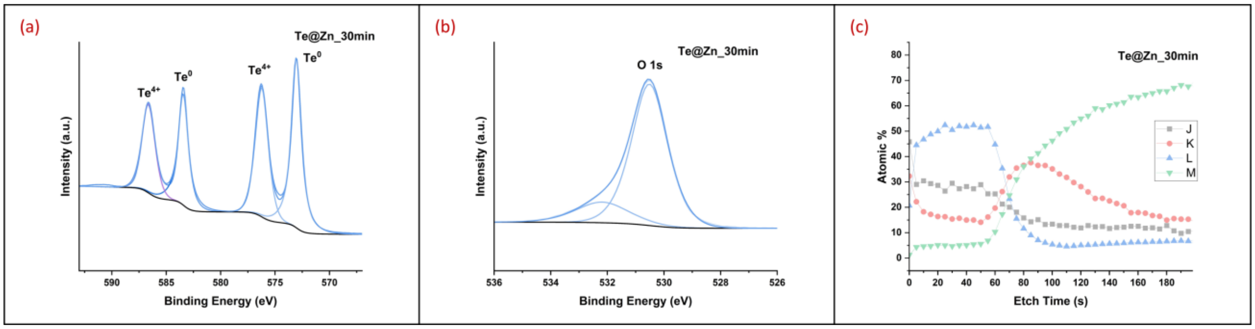


**Figure S3.** High-resolution XPS of (a) Te 3d scan, (b) O 1s scan, and (c) depth profile of the Te@Zn_30 min sample.

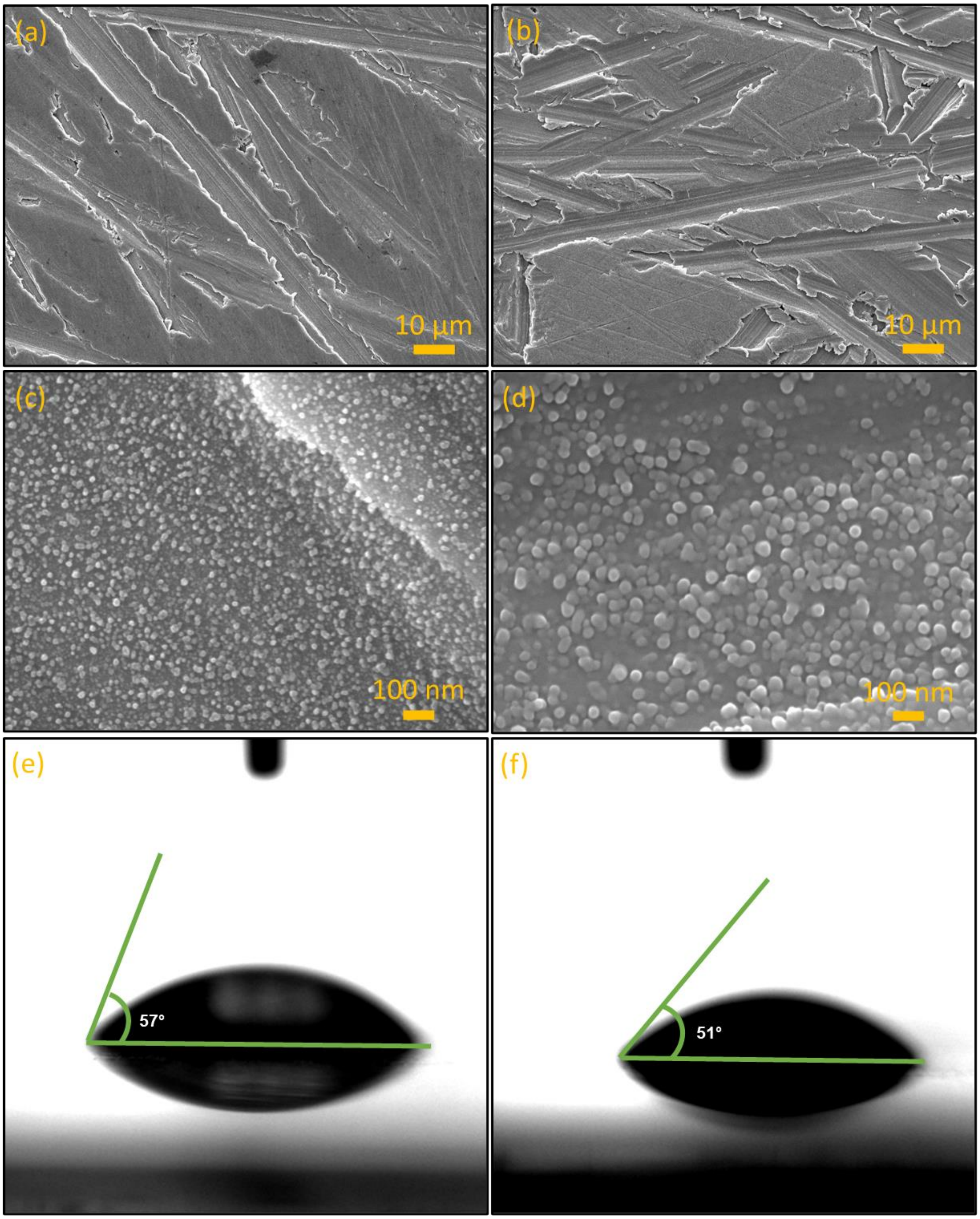


**Figure S4.** Surface morphology and wettability of Zn plates with and without a ~47 nm Te thin film layer. Polished Zn plates before Te deposition: (a) low-magnification SEM, (c) high-magnification SEM, and (e) contact angle (CA) measurement. Polished Zn plates after Te deposition: (b) low-magnification SEM, (d) high-magnification SEM, and (f) CA measurement.
